# Exploring the Impact of 2023 Wildfires on Generalized Anxiety Disorder Symptoms among Residents in Alberta and Nova Scotia

**DOI:** 10.1192/j.eurpsy.2024.867

**Published:** 2024-08-27

**Authors:** G. Obuobi-Donkor, R. Shalaby, B. Agyapong, R. D. L. Dias, V. I. O. Agyapong

**Affiliations:** ^1^Department of Psychiatry, Dalhousie University, Halifax; ^2^Department of Psychiatry, University of Alberta, Edmonton, Canada

## Abstract

**Introduction:**

Raging wildfires are rising in diverse areas, leading to significant environmental and psychological repercussions that are drawing growing concern.

**Objectives:**

This study seeks to assess the prevalence of likely Generalized Anxiety Disorder (GAD) and investigate the factors contributing to its occurrence amidst the wildfires in Alberta and Nova Scotia.

**Methods:**

Data were collected online through a cross-sectional survey from May 14 to June 23, 2023. Alberta and Nova Scotia participants self-subscribe to the program by texting ‘HopeAB’ or ‘HopeNS’ to a designated short code, respectively. The GAD-7 validated scale assessed likely GAD symptoms among the participants.

**Results:**

There were 298 respondents in this study, with a majority residing in Alberta/Nova Scotia areas affected by recent wildfires (62.3%). Among the respondents, 41.9% were likely to experience Generalized Anxiety Disorder (GAD) symptoms. Those living in regions recently impacted by wildfires in Alberta/Nova Scotia were found to be twice as likely to have GAD symptoms, with an odds ratio of 2.4 and a confidence interval of 95% ranging from 1.3 to 4.3.

**Conclusions:**

The study’s findings highlight a relationship between living in areas affected by wildfires and the likelihood of experiencing generalized anxiety disorder (GAD). Exploring potential predictors through additional research could aid in developing strategies to alleviate the mental health impact of natural disasters.

**Disclosure of Interest:**

None Declared

